# Overview on the role of dietary *Spirulina platensis* on immune responses against Edwardsiellosis among *Oreochromis niloticu*s fish farms

**DOI:** 10.1186/s12917-024-04131-7

**Published:** 2024-07-04

**Authors:** Lamiaa A. Okasha, Jehan I. Abdellatif, Ola H. Abd-Elmegeed, Ahmed H. Sherif

**Affiliations:** 1 Bacteriology unit, Animal Health Research Institute AHRI, Agriculture Research Center ARC, Kafrelsheikh, 12619 Egypt; 2 Fish Diseases Department, Animal Health Research Institute AHRI, Agriculture Research Center ARC, Giza, Kafrelsheikh, 12619 Egypt; 3 Aquatic Animal Medicine and Management Department, Faculty of Veterinary Medicine, Cairo University, Giza, 12211 Egypt

**Keywords:** *Edwardsiella tarda*, Nile tilapia, *IL-1β*; *TNF-α*, Fish behavior, *Spirulina*

## Abstract

Edwardsiellosis is a bacterial fish disease that mostly occurs in freshwater farms and is characterized by a high mortality rate. *Edwardsiella tarda* strain was recovered from 17 fish out of 50 Nile tilapia, which were harboring clinical signs of systemic septicemia. The level of un-ionized ammonia (NH_3_) in the fish farm’s water was 0.11–0.15 mg/L, which was stressful for the Nile tilapia.

Sequencing of the *gyrB1* gene confirmed that the isolate was *E. tarda* JALO4, and it was submitted to NCBI under the accession number PP449014. The isolated *E. tarda* harbored the virulence gene *edw1* AHL-synthase (quorum sensing). In addition, the isolate was sensitive to trimethoprim and sulfamethoxazole mean while it was intermediate to florfenicol. The median lethal dose (LD_50_) of *E. tarda* JALO4 was determined to be 1.7 × 10^5^ CFU/mL in Nile tilapia.

In the indoor experiment, Nile tilapia (45.05 ± 0.4 g), which received dietary *Spirulina platensis* (5 and 10 g/kg fish feed), showed optimum growth and feed utilization. Meanwhile, after receiving dietary *S*. *platensis*, the fish’s feed conversion ratio (FCR) was significantly enhanced compared to the control, which was 1.94, 1.99, and 2.88, respectively. The expression of immune-related genes interleukin (*IL)-1β* and tumor necrosis factor *(TNF)-α* were upsurged in *E. tarda-*challenged fish with higher intensity in *S. platensis* groups. Dietary *S. platensis* at a dose of 10 g/kg fish feed could provide a relative protection level (RPL) of 22.2% Nile tilapia challenged against *E. tarda*. Nile tilapia experimentally infected *E. tarda*, drastically altering their behavior: higher operculum movement, low food apprehension, and abnormal swimming dietary *S. platensis* (10 g/kg fish feed) could rapidly restore normal status.

It was concluded that Edwardsiellosis could alter Nile tilapia behavior with a high loss in fish population. Fish received dietary-*S. platensis* could rapidly restore normal behavior after *E. tarda* infection. It is recommended the incorporation of *S. platensis* at doses of 10 g/kg into the Nile tilapia diet to boost their immunity and counteract *E. tarda* infection.

**Supplementary Information:**

The online version contains supplementary material available at 10.1186/s12917-024-04131-7.

## Introduction

Aquatic animals are poikilothermic, and their immune responses are affected by the surrounding environment, such as water parameters salinity, temperature, pH, dissolved oxygen, and ammonia compounds [1, 2]. Nile tilapia *(Oreochromis niloticus)* belongs to the Cichlidae family that originated from Africa, and it can tolerate a wide range of water temperature16–38 °C; beyond this level, health hazards occurred due to deterioration of several metabolic functions resulting in a decrease of feed intake and immune suppression becoming more vulnerable to opportunistic pathogens, even become lethal below 10 °C [3, 4].

Microbial diseases are a major constraint in aquaculture and cause annual fish losses, job casualties, economic crises, and food insecurity; fish pathogens are mainly opportunistic, affecting immune-depressed fish [5–8]. The genus *Edwardsiella* belongs to the Enterobacteriaceae family had five species that are pathogenic to fish: *E. tarda*, *E. ictaluri*, *E. hoshinae*, *E. piscicida* according to **Abayneh et al.** [9], and *E. anguillarum* according to **Shao et al.** [10], *E. tarda* is a Gram-negative bacilli that is known as highly pathogenic bacteria in both humans and aquatic animals [11, 12]. Several studies have shown phenotypic variations and interspecific diversity between isolates retrieved from different geographic regions and host species [13]. Therefore, rapid and precise detection of the causative agent is a principal issue for the control and epidemiological studies [14]. It affects many aquatic species, causing mass mortality and considerable economic losses worldwide [15]. Several reports on the occurrence of Edwardsiellosis in non-catfish species included the Rosy Barb (*Puntius conchonius*) [16], Nile tilapia in the Western Hemisphere [17], and zebrafish *(Danio rerio)* [18].

Circumstances leading to immune suppression are inevitable in aquaculture (open system), so natural immune promoters could manage Nile tilapia to withstand opportunistic pathogens.

*Spirulina platensis* is a microscopic, filamentous, multicellular, and photosynthetic blue-green alga commonly used as a safe and functional supplement in animal feed. *S. platensis* possesses a smooth body with a delicate cell wall, making it highly digestible [19, 20]. *S. platensis* is a nutritionally dense cyanobacteria containing appreciable levels of amino acids, fatty acids, vitamins, minerals, pigments, and digestive enzymes that are considered beneficial to health and improve fish resistance against pathogens and environmental stressors [21–23]. *S. platensis* has two distinctive antioxidant pigments (carotenoids and phycocyanin) and vitamins (E and C), in addition to chlorophyll, fucoxanthin, polyphenols, and polysaccharides [23, 24]. *S. platensis* enhanced nonspecific immunity of channel catfish *(Ictalurus punctatus)* and increased host defense mechanisms [25], **Nile tilapia** [26]. Meanwhile, **Watanuki et al.** [27] found that dietary *S. platensis* could stimulate the immune system of common carp *(Cyprirus carpio).*

In this work, we investigate the bacterial pathogen that was accompanied by high mortality in freshwater fish farms, the alteration in fish behavior and immune responses associated with bacterial infection, and the possible amelioration role of *S. platensis.*

## Materials and methods

### Case history and sampling

Nile tilapia *(Oreochromis niloticus)* weighing 80–120 gm were cultured in earthen ponds at a density of 30,000 per acre; paddlewheel aerators were not used on the farm. Fish experienced approximately 50 deaths/acre/day during the early summer of 2023 on a private farm in Kafrelsheikh governorate, Egypt. Moribund fish had low feed intake, anorexia, abnormal swimming behavior, lethargy, skin ulcerations, and hemorrhage.

Moribund and dead Nile tilapia from the affected fish farm were transferred to the bacteriology laboratory of fish diseases at the Animal Health Research Institute (AHRI) within 1–2 h using an ice box. The fish were examined for clinical signs and post-mortem lesions of fish diseases [28].

### Examination of fish farm water

On fish farm, physical water parameters were determined including water temperature and salinity (model YSI environmental, EC300), dissolved oxygen (DO) (Aqualytic, OX24), and pH (Thermo Orion, model 420 A).

Samples of farm water were collected in septic polyethylene bottles to determine the concentration of ammonia compounds including: total ammonia nitrogen (TAN), unionized ammonia (NH_3_), nitrite (NO_2_), and nitrate (NO_3_), samples were transferred on ice to the laboratory of Animal Health Research Institute (AHRI), and were analyzed using a UV/Visible spectrophotometer, Thermo-Spectronic 300 [29].

### Bacteriological examination

#### Isolation, phenotypic and biochemical identification of *Edwardsiella spp*

The preliminary identification of bacterial isolates: Swabs were taken from the Nile tilapia organs, kidney, heart, liver, and spleen, then cultivated into tryptic soy broth (TSB) at 30 °C/24 h. Loopfuls from the bacterial broth culture were cultivated onto agar tryptic soy with 5% sheep blood and incubated at 30 °C/24 h. Prevailed colonies were randomly selected and purified by sub-culturing onto TSA. The isolated colonies were sub-cultivated onto selective agar Salmonella-Shigella agar. Bacterial isolates were stored at -80 °C in tryptic soy broth with glycerol for further analysis. The isolate was identified using the Gram-stain and API 20E (bio-Merieux) [30, 31]. All bacterial media are manufactured by Difco, Detroit, USA.

#### Molecular identification of Edwardsiella spp

##### DNA extraction and polymerase chain reaction PCR

To identify the bacterial isolate, the bacterial DNA was extracted and examined for the presence of the *E. tarda gyrB1* gene. It was also tested for virulence-determinant genes, including *Cds1* chondroitinase, *edwI* AHL-synthase, *qseC* sensor protein, and *pvsA* vibrioferrin synthesis. The extraction process of bacterial DNA was performed using the PathoGene-spin™ DNA Extraction Kit (iNtRON Biotechnology, Seongnam, Korea). Briefly, the PCR reaction consisted of a 25 µl mix containing 12.5 µl of 2X MyTaq HS mix (Bioline, Meridian Life Science, UK); 1 µL of each primer, 100 ng of template DNA; and RNase-free water. The PCR products were separated using electrophoresis on agarose gel 1% (Applichem, Germany, GmbH) and were photographed by a gel documentation system (Alpha Innotech, Biometra). The primers and PCR conditions for gene amplification are in Table 1.

##### Sequencing for strain identification

The extracted DNA was adjusted to 50 ng/µl and then stored at − 20 ˚C for sequencing. The isolate of *Edwardsiella* strain was identified and discriminated based on DNA sequencing analysis of *E. tarda gyrB1.* The amplicons were excised from the gel and purified using the QIAquick extraction kit (Qiagen, Germany). Nucleotide sequencing of the amplified genes was performed using the Big Dye terminator v3.1 kit (Life Technologies, Applied Biosystems, Foster City, CA, USA) by ABI 3730xl DNA sequencer (Applied Biosystems™, USA) using a primer of the PCR reaction in both directions. The raw sequence was manually checked and edited for ambiguities using a sequence alignment editor (BioEdit v. 7.2.5) [32]. Finally, the edited sequence was submitted to the GenBank database to get the accession number.

##### Phylogenetic tree

The Neighbor-Joining phylogenetic tree was conducted to match the *gyrB1* of *Edwardsiella* spp. with the accession numbers of typing strains retrieved from the GenBank database using MEGA X [33]. The phylogenetic tree of *sodB* gene was rooted on *Vibrio anguillarum* strain ATCC 43,314 (KU755359.1), which was used as an outgroup. The lineage supports were assessed using nonparametric bootstrapping with 1000 replicates. The following factors were also employed: substitutions: transversions and transitions; pattern among lineages: homogeneous; and 95% cutoff partial deletion principal.

##### Gene expression of immune-related genes

The impact of dietary ***S. platensis*** on the expression of pro-inflammatory genes in the head kidney of the experimental Nile tilapia. The interleukin *(IL)-1β* and tumor necrosis factor *(TNF)-α* genes (Table 1) was assessed using quantitative real-time polymerase chain reaction (RT-PCR). The RNA was extracted with Trizol reagent (iNtRON Biotechnology Inc., Korea), and samples were collected from three Nile tilapia, each group using Nanodrop D-1000 spectrophotometer (NanoDrop Technologies Inc., USA). The obtained RNA was assessed for quality and quantity and kept at − 80 °C. The β-actin was used as a housekeeping gene. The results were assessed using Eq. 2^−ΔΔCT^ [34].

**Table 1:** Primers sequences, target genes, amplicon sizes and cycling conditions

Target gene	Function	Sequences	Size (bp)	Annealing ˚C/sec	Reference
***gyrB1***	ATPase domain	F: GCATGGAGACCTTCAGCAAT R: GCGGAGATTTTGCTCTTCTT	415	50/40	[35]
***Cds1***	Chondroitinase	F: TCTCCACCCATAATGCCACG R: CAAACGGCGTCGTGTAGTCG	435	55/40	[36]
***edwI***	AHL-synthase	F: ATCCGCAGCATCGAATGGCT R: GAAGGATAACGATGTGGTGT	360	55/40	
***qseC***	sensor protein	F: CAGCAGTAGCAGGATCACCA R: ATGGACGTATGCTGCTCAAC	260	55/40	
***pvsA***	vibrioferrin synthesis	F: CTGGAGCAGTACCTCGACGG R: CGATGCTGCGGTAGTTGATC	313	55/40	
***β-actin***	House keeping	F: AGCAAGCAGGAGTACGATGAG R: TGTGTGGTGTGTGGTTGTTTTG	135	58.5/30	XM_003443127.5
***IL-1β***	Pro-inflammatory	F: T GCTGAGCACAGAATTCCAG R: GCTGTGGAGAAGAACCAAGC	172	60/30	XM_019365841.2
***TNF-α***	Pro-inflammatory	F: CCAGAAGCACTAAAGGCGAAGA R: CCTTGGCTTTGCTGCTGATC	82	59.9/30	AY428948.1

#### Lethal median dose and pathogenicity testing

The LD_50_ was determined briefly according to the procedure described by **Reed and Muench** [37], briefly. After acclimatization at the wet laboratory of the Animal Health Research Institute, Nile tilapia (*n* = 10) with a mean weight of (70.8 ± 0.3 g) were anesthetized with 150 µg/ L of tricaine methanesulfonate (MS222; Sigma, St. Louis, MO, the USA), then fish were injected. Ten fish (duplicate groups) were intraperitoneally injected with 100 µl suspension of serial tenfold dilutions of *E. tarda* 24 h-old culture (1 × 10^2^, 1 × 10^3^,1 × 10^4^, 1 × 10^5^, 1 × 10^6^, 1 × 10^7^, 1 × 10^8^, 1 × 10^9^, or 1 × 10^10^ CFU/mL). Mortality rates were recorded for 14 days, and *E. tarda* was re-isolated from the dead moribund fish and confirmed via PCR.

#### Antimicrobial sensitivity analyses

The susceptibility of isolated *E. tarda* strain to antimicrobial agents was determined using the disc diffusion method [38]. The bacteria was cultured onto Mueller–Hinton agar (Oxoid™) for 24 h at 30 °C, then discs were seeded on the agar, discs were containing tetracycline 30 µg, trimethoprim/sulfamethoxazole (SXT) 1.25/23.75 µg), ciprofloxacin (CIP) 5 µg, florfenicol 30 µg, erythromycin (E, 15 µg), gentamicin 10 µg, amoxicillin 10 µg, ampicillin (AMP) 10 µg, kanamycin 30 µg, cefotaxime 30 µg, and streptomycin 30 µg). The results were calculated by measuring the inhibition zones [38]. Also, the multi-drug resistance (MDR) index using the following equation [39],

$$\text{M}\text{D}\text{R} =\frac{X}{\text{Y}}$$

Where X is the number of antibiotics that the isolate was resistant to and Y is the number of antibiotics used, if the MDR index is higher than 0.2, the bacterial isolate is multiple antibiotic-resistant.

### Feeding trial

In an indoor trial to improve the performance of Nile tilapia to combat the recovered pathogens, the green algae *Spirulina platensis* was added to the fish diet. Nile tilapia was collected from freshwater fish farm with no history of high mortality or health troubles. Fish weighing 45.05 ± 0.4 g was tranquilized in the fish farm and then swiftly transferred in labeled plastic bags filled with 1/3 clean water and 2/3 pure oxygen. At the farm site, fish were tranquilized using anesthetic agent MS-222 (tricaine methanesulfonate) at a dose of 40 mg/L of transporting water; MS-222 is produced by Syndel, Canada. Fish were subjected to an iodine bath at 20 ppm/10 minutes (Betadine®, the active ingredient, is 5% of povidone-iodine and produced by the Nile Company for Pharmaceuticals) at the arrival in the wet laboratory at Animal Health Research Institute, Agriculture Research Center, Kafrelsheikh Egypt [40, 41].

All aquaria were well aerated via an air bubble stone diffuser connected with a plastic hose and an air blower. One-third of water was replaced with clean, fresh water day after day to maintain constant water parameters of the aquaria; temperature (27 ± 1.2 °C), salinity (0.2 ± 0.05 g/L), pH (7.2 ± 0.2), and DO (5.2 ± 0.3 mg/L), TAN (0.09 ± 0.02 mg/L), NH_3_ (0.02–0.01 mg/L), NO_2_ (0.00 mg/L), and NO_3_ (0.5 ± 0.01 mg/L).

With meticulous precision, the fish food (in pellet form) was soaked and fully homogenated until a paste was formed. The gelatin (Canal AquaCure, Egypt) was then added to the feed paste/*S. platensis* mixture, enhancing the feed’s consistency. This mixture was left to dry at room temperature and subsequently cut into small, uniform sizes.

The feeding trial lasted for four weeks prior to the experimental infection. Fish feed was offered at 0.9.00 a.m. and 03.30 p.m. at a rate of 5% b.w. fish per day, with a basal diet composition of crude protein of 30.95% and digestible energy of 3400.5 kcal/kg. The diet was purchased from the local market and manufactured by Aller Aqua® Egypt (Reg. no. 9782). Lot. no. 365,622 https://www.aller-aqua.com/.

The variation in the feeding rate of the fish’s body weight was adjusted weekly. The growth performance and feed utilization were determined using the following equations,

$$\text{W}\text{G}=\text{F}\text{W} - \text{I}\text{W}$$

$$\text{D}\text{W}\text{G}=(\text{F}\text{W}- \text{I}\text{W})/\text{t}$$

$$\text{F}\text{C}\text{R}=\text{F}\text{I}/\text{W}\text{G}$$

Where final body weight (FW), initial body weight (IW), daily weight gain (DWG) feed conversion ratio (FCR), and t = time (days).

### Experimental infection

To examine the pathogenicity of isolated *E. tarda*, 15 fish experimental fed *S. platensis* and control (control positive) were injected (I/P) with 0.1 mL of a bacterial solution containing LD_50_ 1.7 × 10^5^ CFU/mL, the bacteria dose was adjusted to LD_50_ using the McFarland scale in phosphate buffer saline. At the same time, a negative control group was schooled by injecting 15 fish with pure saline 0.65 mg/l in concentration (control negative) [42]. Each dead fish was counted if *E. tarda* was re-isolated for 14 days. the mortality rate (MR) and relative protection level RPL [43] were estimated using the following equations:

$${\text{MR}}\% {\text{ = }}\frac{{{\text{number of deaths in a specific period}}}}{{{\text{total population during that period}}}} \times {\text{100}}$$

$${\text{RLP}}\% {\text{ = }}({\text{1 - }}\frac{{\% {\text{deaths in the treated group}}}}{{\% {\text{deaths in the control group}}}}) \times {\text{100}}$$

Bacteria were re-isolated following the scheme in the above section. The phenotypic profiles of *E. tarda* were done according to Bergey’s recommendations [44], and biochemical characters were illustrated using API 20E strips [45].

### Fish reflexes

Fish breath was assessed by operculum movement (OM), which was recorded by visually counting the number of *opercular beats* for 1 min at one-h intervals [46]. Scores classify feeding, capture indicators, and respected characteristics for on-farm tilapia welfare assessment [47]. Food apprehension was scored into four levels: 1: in 3: in ≤ 120 s, 2: in 120–180 s, 180–300 s, and 4: No or ≥ 360 s. Escape reflex was scored into four levels 1–4, 1: Normal swimming, 2: Excited swimming behavior, > 20 dorsal fins or low body parts on surface, 3: Swimming in different directions or low activity, and 4: Many fish floating on side and exhausted.

### Histopathological examination

At the end of the feeding trial and bacterial challenge, the gills, liver, and spleen specimens were taken and preserved in buffered formalin (10%) for histopathological analyses. Specimens were dehydrated with alcohol and cleared with xylol. They were embedded in paraffin wax and cut into 4 μm sections. Specimens were stained with hematoxylin-eosin and examined using a light microscope [48].

### Statistical analyses

Data concerning the ameliorating role of *S. platensis* to Edwardsiellosis in the experimental Nile tilapia were analyzed to determine the variance (ANOVA). Significance between the experimental groups using one-way ANOVA test and data were expressed as mean ± standard error (SE) at the significant difference of *P* ≤ 0.05 (the SPSS software for Windows, SPSS Inc., Chicago, IL, USA).

## Results

### Water parameters and natural infection

On the affected fish farm, water parameters were suitable for tilapia culture, including temperature (25.8–27.5 °C), salinity (1.23–1.41 g/L), pH (8.4–8.9), and DO (3.5–5.3 mg/L). Slight increase in ammonia compounds TAN (0.98–1.29 mg/L), NH_3_ (0.11–0.15 mg/L), NO_2_ (0.04–0.06 mg/L), and NO_3_ (0.88–0.93 mg/L).

**Fig. 1:**
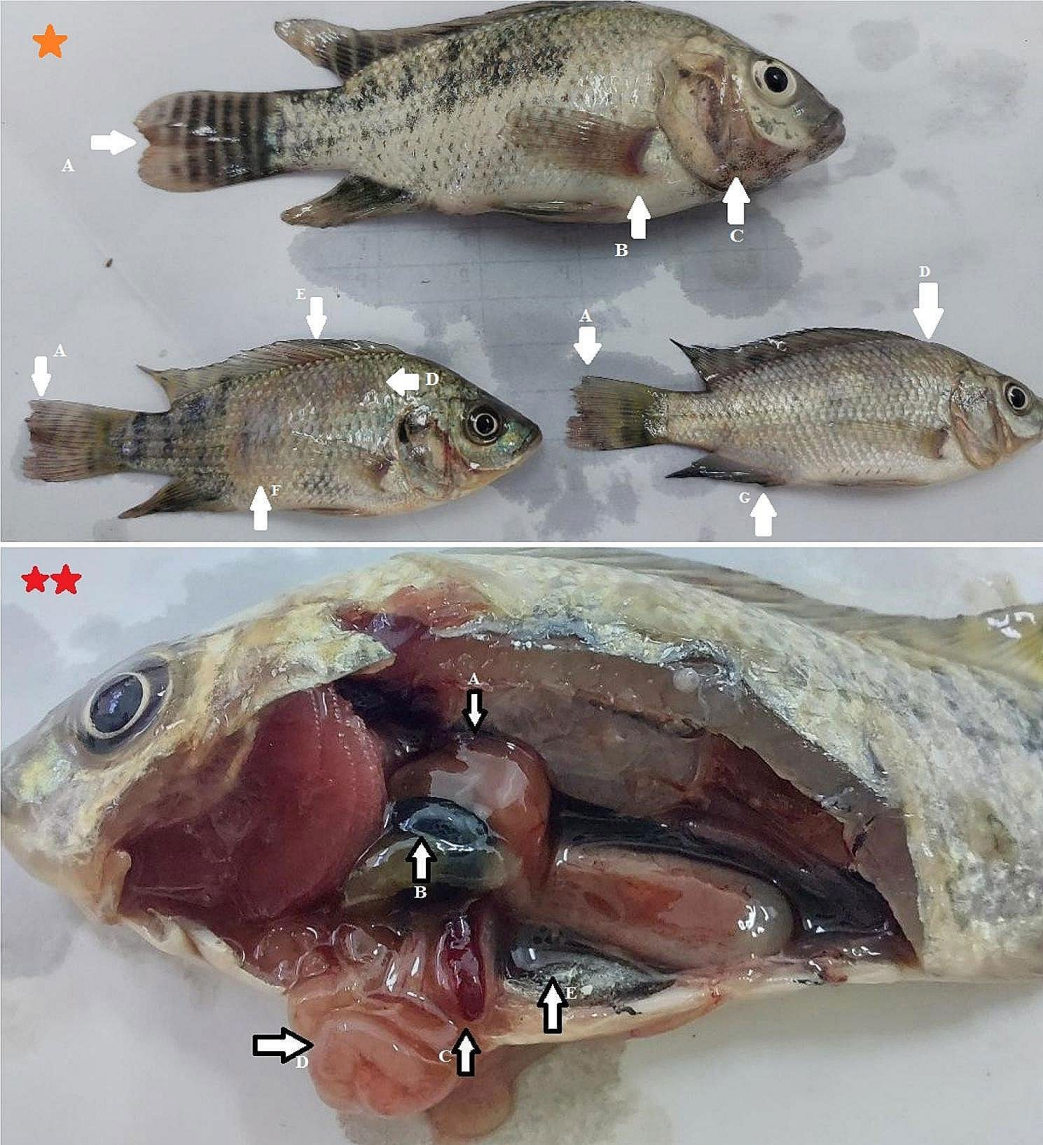
Nile tilapia naturally infected with E. tarda. *(star): **A**; Tail rot, **B**; hemorrhage at base of operculum fin, **C**; hemorrhage at base of anal fin, **D**; hemorrhage at dorsum, E; hemorrhage at base of dorsal fin, F; hemorrhage at the abdomen. ** (2 stars): **A**; brownish red liver, **B**; enlarged gall bladder, **C**; slightly splenomegaly, **D**; slight inflamed intestine, **E**; remnant of abdominal fluid

It was recorded that out of 50 Nile tilapia harboring clinical signs, 17 fish were infected with *E. tarda.* Clinical signs were low feed intake, external hemorrhages on the body surface and fin bases, and dentated tail fin (tail rot), whereas postmortem signs were enlarged brownish-red liver, distended gall bladder, splenomegaly, and inflamed intestine with ascetic fluids (Fig. 1).

### Bacteria identification and median lethal dose LD_50_

By using API 20E, 17 out of 50 Nile tilapia were positive for *E. tarda*, which was identified using API20 E with the identification number (4,144,000), a unique identifier that aids in the classification and tracking of this bacterium.

**Fig. 2:**
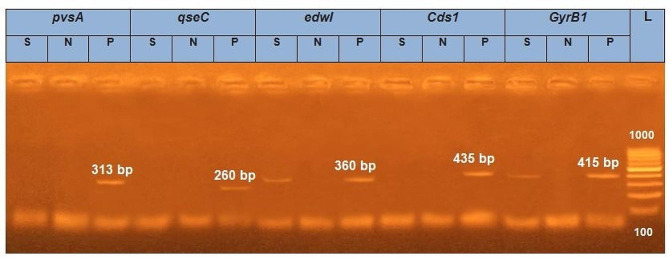
*Edwardsiella tarda* gel electrophoresis *gyrB1* and virulence genes of *E. tarda* JALO4. S; sample, N; negative, L; DNA ladder

**Fig. 3:**
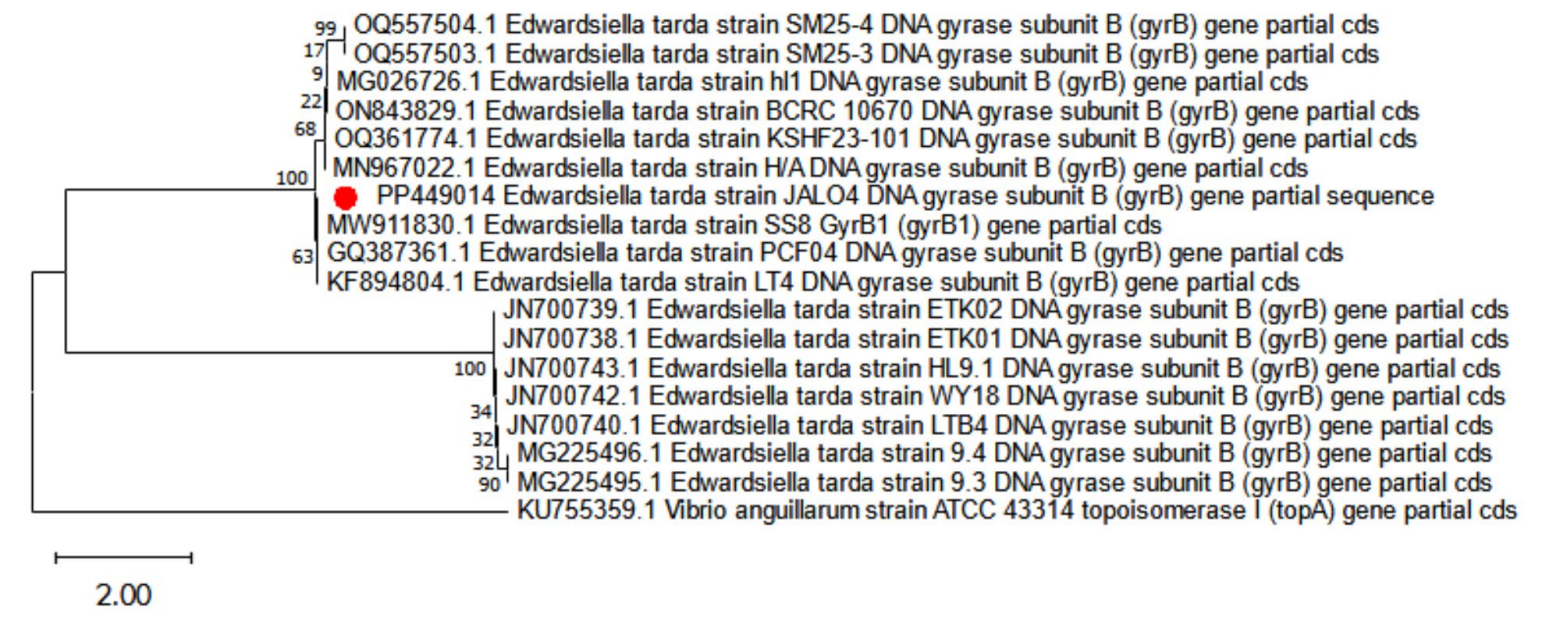
Phylogenetic tree of *gyrB1* gene *E. tarda* JALO4 (PP449014)

In Fig. 2, PCR analyses, the isolated *E. tarda* was examined for the presence *gyrB1*, virulence genes *Cds1, edw1, qseC*, and *pvsA, E. tarda* was positive for *gyrB1* and *edw1* AHL-synthase. The amplified *gyrB1* gene was successfully performed from the *Edwardsiella* isolate using the gene-specific primers pair. The purified PCR products were then sequenced to confirm the identities of bacterial spp. at the species level (Fig. 3), and the isolated strain was identified as *E. tarda* JALO4 under the accession numbers PP449014 were issued by the GenBank staff. The BLAST analysis of *gyrB1* gene sequences proved that bacterial strains belonged to the genus *Edwardsiella*, and was identified as *E. tarda*. The comparative sequence analysis of the current *gyrB1* sequences (PP449014) revealed a high similarity index between 100% and 99.59% with relevant sequences of *E. tarda* (MW911830.1 and GQ387361.1). The alignment of these accession numbers (ON843829.1, MN967022.1, KF894804.1, JN700743.1, JN700742.1, JN700740.1, JN700739.1, JN700738.1, OQ361774.1, OQ557504.1, OQ557503.1, MG026726.1, MG225496.1, and MG225495.1) showed low similarity than 98% *gyrB1* of *E. tarda.* In this study, the typing strain of *Vibrio anguillarum* ATCC 43,314 (KU755359.1) served as an outgroup.

To determine LD_50_, Nile tilapia weighting 50 g were subjected to descending ten-fold dilutions of the isolated *E. tarda* JALO4. The LD_50_ was found to be 1.7 × 10^5^ CFU/mL at water temperature 27.5 ± 2.5 °C, salinity 0.23 g/L, pH 7.9, and DO 5.8 ± 1.5 mg/L.

In Table 2, the isolated *E. tarda* was sensitive to trimethoprim 1.25 µg & sulfamethoxazole 23.75 µg and intermediate to florfenicol 30 µg, meanwhile it was resistant to tetracycline, ciprofloxacin, erythromycin, gentamycin, amoxicillin, ampicillin, kanamycin, cefotaxime, and streptomycin. These findings indicate a significant challenge in treating *E. tarda* infections due to its resistance to a wide range of commonly used antibiotics. The obtained MDR was 0.8, indicating that *E. tarda* JALO4 is a multiple antibiotic-resistant bacteria.

**Table 2:** Data on bacterial strains, accession number, site, virulence genes, and multidrug resistant genes

Antibiotic	Sensitive	Intermediate	Resistant
Tetracycline 30 µg	-	-	+
Trimethoprim 1.25 µg Sulfamethoxazole 23.75 µg	+	-	-
Ciprofloxacin 5 µg	-	-	+
Florfenicol 30 µg	-	+	-
Erythromycin 15 µg	-	-	+
Gentamycin 10 µg	-	-	+
Amoxicillin 30 µg	-	-	+
Ampicillin 10 µg	-	-	+
Kanamycin 30 µg	-	-	+
Cefotaxime 30 µg	-	-	+
Streptomycin 30 µg	-	-	+

*Note* +/-; positive/negative

### Indoor experimental feeding trial

#### Growth performance

In Table 3, Nile tilapia (45.05 ± 0.4 g) were supplemented with dietary *S. platensis* at a dose of 5 and 10 g/kg diet. The growth parameters FW (56.87 and 57.46 g), WG (11.67 and 12.23 g), DWG (0.42 and 0.44 g), and FI (12.2 and 23.66 g) were significantly increased in fish that received dietary *S. platensis* compared to the control 51.2 g, 6.53 g, 0.23 g, and 18.73 g, respectively. A significant enhancement of feed utilization was achieved in *S. platensis* groups as FCR was 1.94 and 1.99 compared to the control 2.88.

**Table 3:** Feeding trial and growth performance of the experimental Nile Tilapia. (mean ± standard error)

Item	Control	S. platensis (5 g)	S. platensis (10 g)
**IW**	44.7 ± 1.15	45.2 ± 1.48	45.2 ± 1.4
**FW**	51.2^**B**^ ± 0.7	56.87^**A**^ ± 1.03	57.46^**A**^ ± 1.33
**WG**	6.53^**B**^ ± 0.6	11.67^**A**^ ± 0.6	12.23^**A**^ ± 0.6
**DWG**	0.23^**B**^ ± 0.02	0.42^**A**^ ± 0.02	0.44^**A**^ ± 0.02
**FCR**	2.88^**A**^ ± 0.11	1.99^**B**^ ± 0.06	1.94^**B**^ ± 0.06
**FI**	18.73^**B**^ ± 1.05	23.2^**A**^ ± 1.66	23.66^**A**^ ± 0.5

*Note* Sp5; *S. platensis* 5 g/ kg fish feed, Sp10; *S. platensis* 5 g/ kg fish feed. Different letters indicate significant difference at *P* ≤ 0.05 in the same row

#### The expression of immune-related genes

**Fig. 4:**
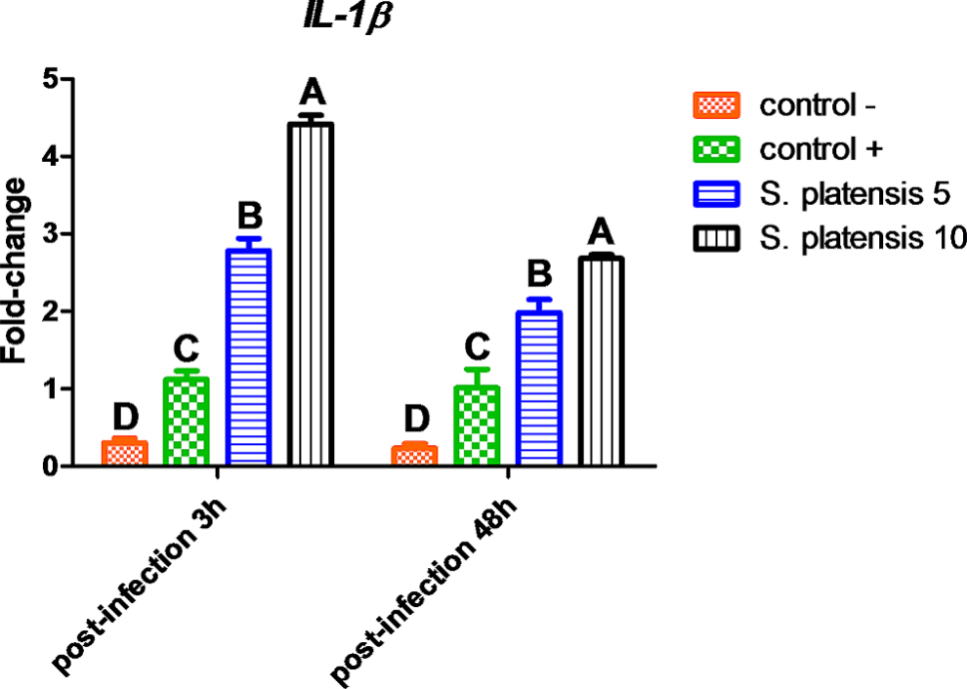
Gene expression of *IL-1β* in the head kidney of Nile tilapia experimental infected with *E. tarda.* Different letters indicate significant difference at *P* ≤ 0.05. (-); negative control fish were injected with saline, **(+)**; positive control fish were injected with *E. tarda*

**Fig. 5:**
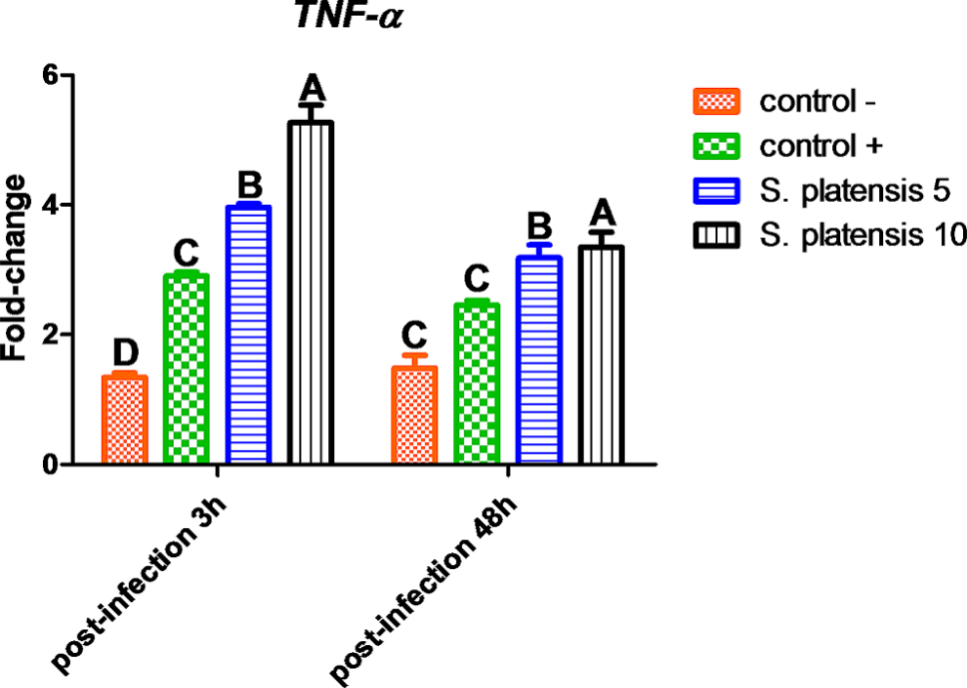
Gene expression of *TNF-α* in the head kidney of Nile tilapia experimental infected with *E. tarda.* Different letters indicate significant difference at *P* ≤ 0.05. (-); negative control fish were injected with saline, **(+)**; positive control fish were injected with *E. tarda*

Gene expression of pro-inflammatory *IL-1β* (Fig. 4) and *TNF-α* (Fig. 5) were affected by both dietary *S. platensis* and *E. tarda* infection. Post-infection by 3 h, *IL-1β* expression was significantly upsurged with *E. tarda* infection 2.78 and 4.42 fold-change in the head kidney of Nile tilapia received *S. platensis* at a dose of 5 g/kg fish feed and 10 g/kg fish feed, respectively, compared to infected control positive (+) and uninfected control negative (-) 1.12 and 0.3 fold-change, respectively. Following the trend of *IL-1β* expression, *TNF-α* expression was upsurged in response to *E. tarda* infection in fish that received *S. platensis* at doses of 5 g and 10 g/kg fish feed compared to infected control.

After 48 h post-infection, the gene expression of *IL-1β* and *TNF-α* expression significantly decreased compared with 3-h post-infection.

#### Experimental infection and relative protection level of *S. platensis*

**Fig. 6:**
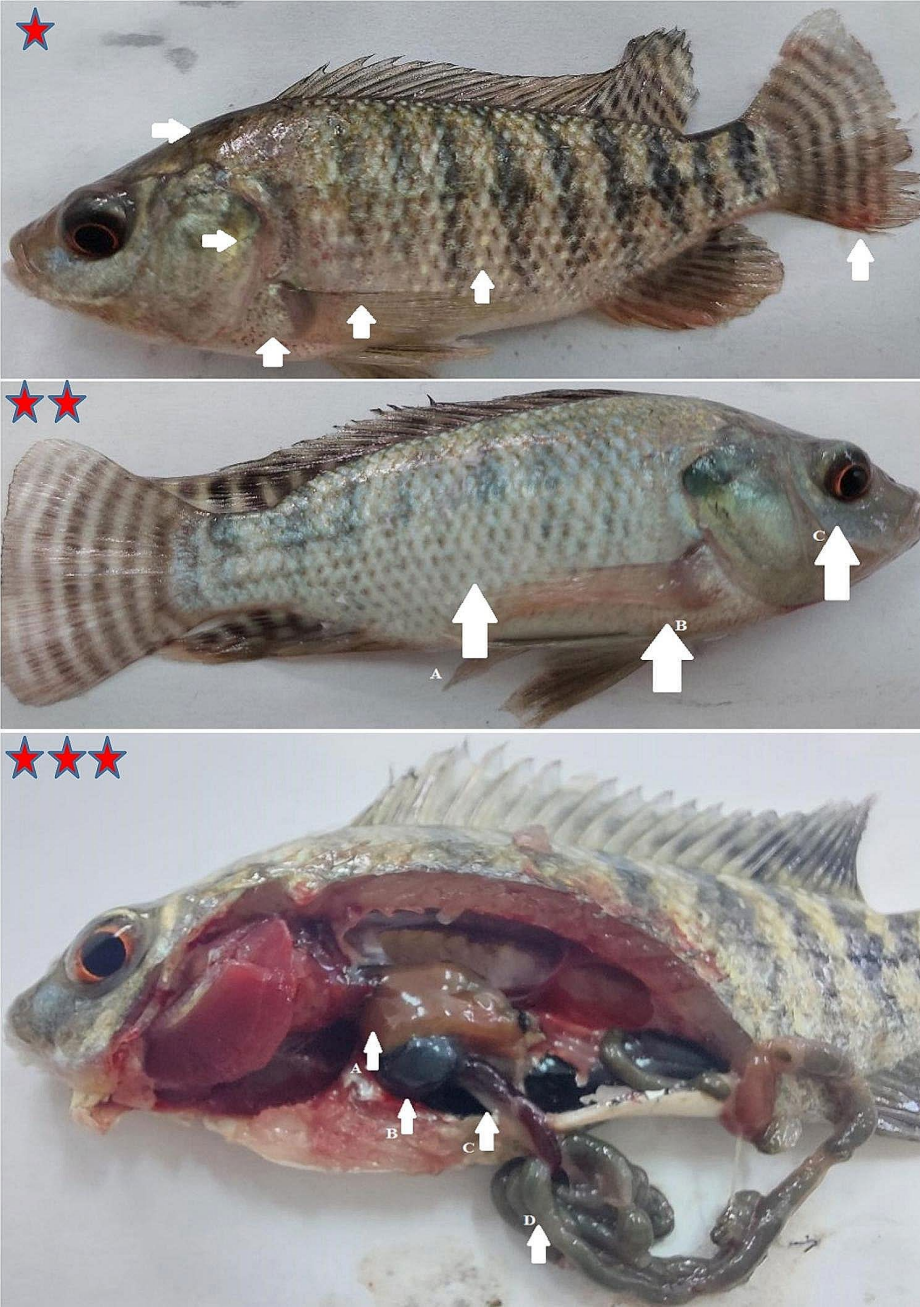
Nile tilapia experimentally infected with E. tarda. *(star): multiples hemorrhages on external body surface. ** (2 stars): **A**; body discoloration paleness, **B**; hemrrahges at base of operculum fin, **C**; pop-eye. *** (3 stars): **A**; brownish liver with hemorrhage on surface, **B**; enlarged gall bladder, **C**; splenomegaly, **D**; intestine with greenish content

In Fig. 6, clinical signs of the experimentally infected Nile tilapia were similar to those obtained with naturally infected ones: external body discoloration (paleness), scale loss, and slight exophthalmia. Also, post-mortem were brownish hemorrhagic liver, distended gall bladder, splenomegaly, and a partially empty intestine with greenish content.

In Table 4, MR% was decreased in the fish group that received *S. platensis* at a dose of 10 g/kg fish feed compared with the control and those that received 5 g /kg fish feed: 46.67%, 60%, and 60%, respectively. Supplementation of Nile tilapia with *S. platensis* at a dose of 10 g/kg fish feed could provide a RPL of 22.2%.

**Table 4:** Mortality rate of Nile tilapia experimentally infected with *E. tarda* JALO4 and *S. platensis* treatment

Item	Control (-)	Control (+)	S. platensis (5 g)	S. platensis (10 g)
**Fish no.**	15	15	15	15
**Deaths**	2	9	9	7
**Survived**	13	6	6	8
**MR%**	13.33	60	60	46.67
**RPL%**	-	-	0	22.2

*Note* no; number, (-); negative control fish were injected with saline, **(+)**; positive control fish were injected with *E. tarda*

### Fish behavioral reflexes at 3 and 14 days post-infection

In Table 5, Nile tilapia experimentally infected with *E. tarda* showed drastic behavior changes; OM (opercular beats for 1 min period at 1 h interval) was increased regardless *of S. platensis* supplementations. Meanwhile, OM was higher in fish that received dietary *S. platensis* 55–61 opercular beats in the 3 days post-infection, restoring average values in 14 days compared to the control. Food apprehension was decreased in response to *E. tarda* infection, and *S. platensis* supplementation could enhance and restore average values after 14 days post-infection. Nile tilapia experimentally infected with *E. tarda* lost their normal swimming behavior, and dietary *S. platensis* could reserve normal behavior in the 3 days post-infection; 14 days post infections survived, and fish could normally swim.

**Table 5:** Fish behavioral reflexes at 3 and 14 days post-infection

Groups		Control (-)	Control (+)	S. platensis (5 g)	S. platensis (10 g)
**Operculum movement**	**3 days**	42–48	54–55	59–61	55–60
	**14 days**	**42–46**	**39–40**	**47–48**	**47–49**
**Food apprehension**	**3 days**	3	3–4	2–3	2–3
	**14 days**	**2**	**3**	**1–2**	**1–2**
**Capture**	**3 days**	1	4	2–3	2–3
	**14 days**	**1**	**1**	**1**	**1**

*Note* (-); negative control fish were injected with saline, **(+)**; positive control fish were injected with *E. tarda*

### Histopathological analyses pre- and post-infection

**Fig. 7:**
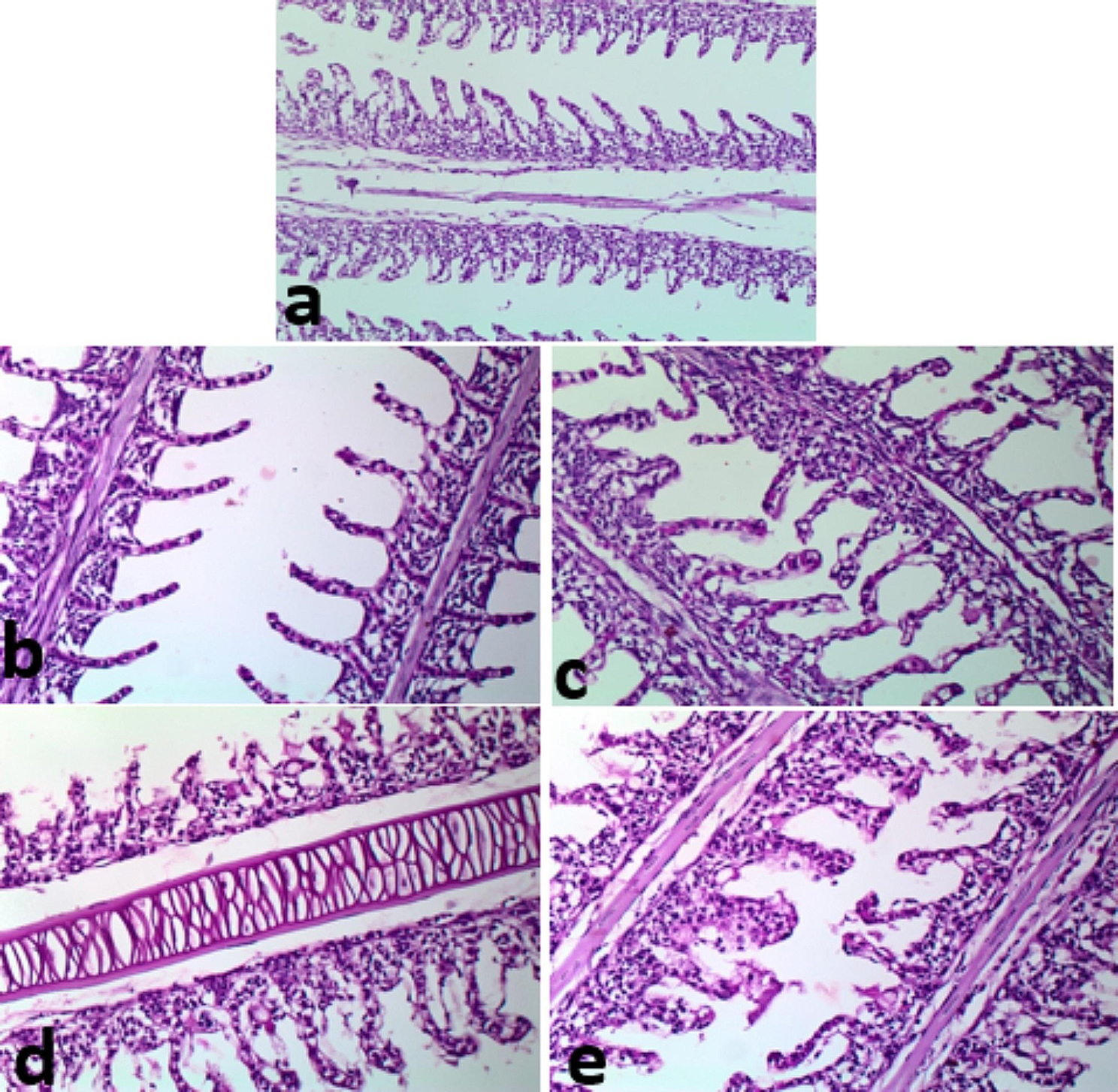
Normal histological criteria of gills in (**a**, **b**& **c**), with truncated lamellae, some showing tips sloughing (**d**), lamellar atelectasis (lamellar aneurysm) in (**e**). Note: (**a**); control fish, (**b**); fish received 5 g/kg fish feed *S. platensis*, (**c**); fish received 10 g/kg fish feed *S. platensis*, (**d**); control infect with *E. tarda*, (**e**); fish received *S. platensis* and infect with *E. tarda.* H&E X 400

**Fig. 8:**
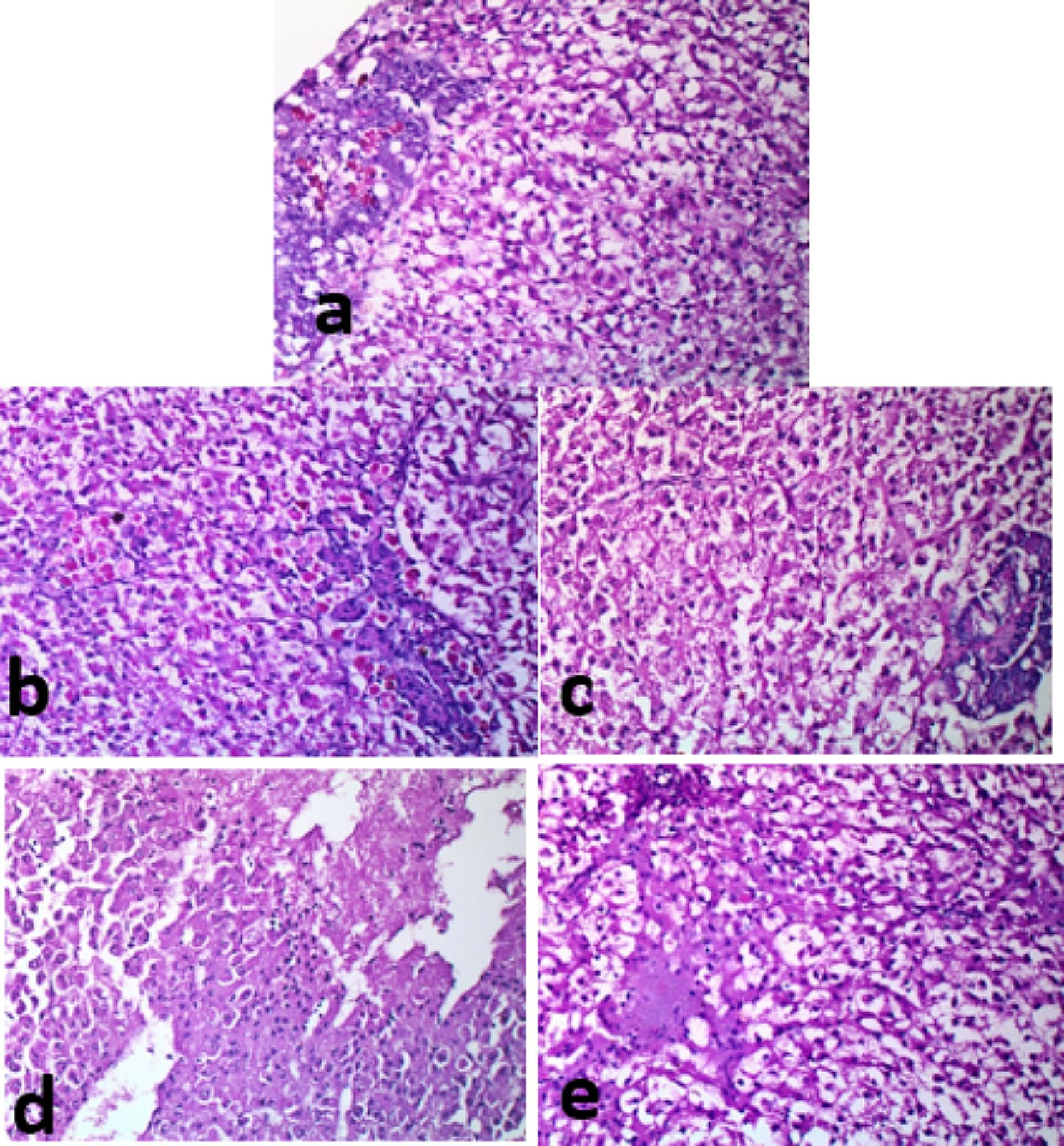
Liver with evidence of liquefactive necrosis in the form of a wide area of lysed hepatic- parenchyma (**d**), normal hepatic parenchyma, development of pancreatic acini (**a**, **b** &**c**) with occurrence of some melano-macrophage cells (**a**&**b**). Small lysed area still detected in hepatic parenchyma (**e**) *Note* (**a**); control fish, (**b**); fish received 5 g/kg fish feed *S. platensis*, (**c**); fish received 10 g/kg fish feed *S. platensis*, (**d**); control infect with E. tarda, (**e**); fish received *S. platensis* and infect with *E. tarda.* H&E X 400

**Fig. 9:**
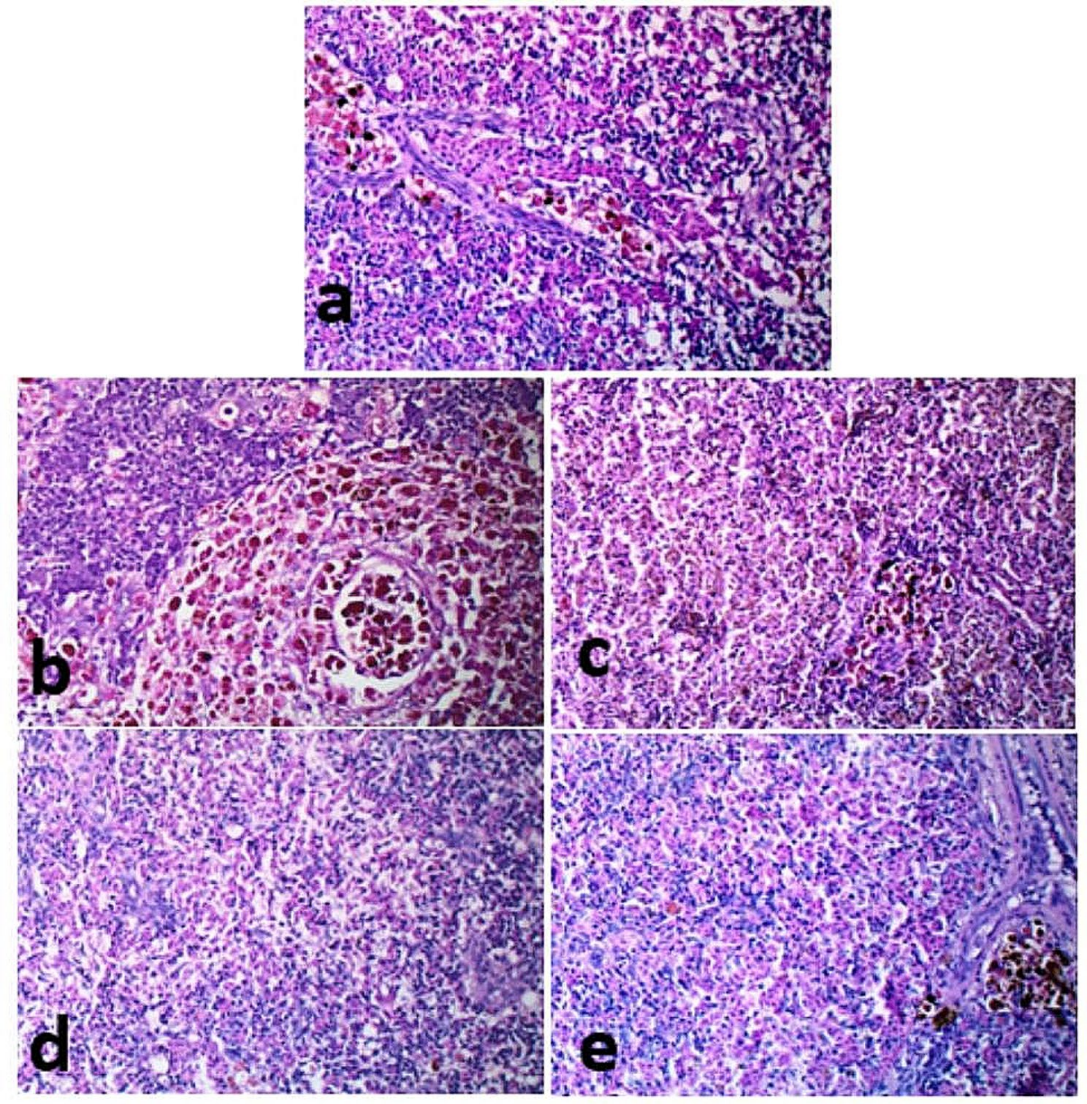
Spleen parenchyma revealing moderate melano-macrophage centers (**a** & **e**), wide-sized foci of melano-macrophage (**b**), dispersed melano-macrophage cells (**c**), and marked depletion of lymphocytic elements with absence of melano-macrophage centers (**d**). *Note* (**a**); control fish, (**b**); fish received 5 g/kg fish feed *S. platensis*, (**c**); fish received 10 g/kg fish feed *S. platensis*, (**d**); control infect with E. tarda, (**e**); fish received *S. platensis* and infect with *E. tarda.* H&E X 400

**Fig. 10:**
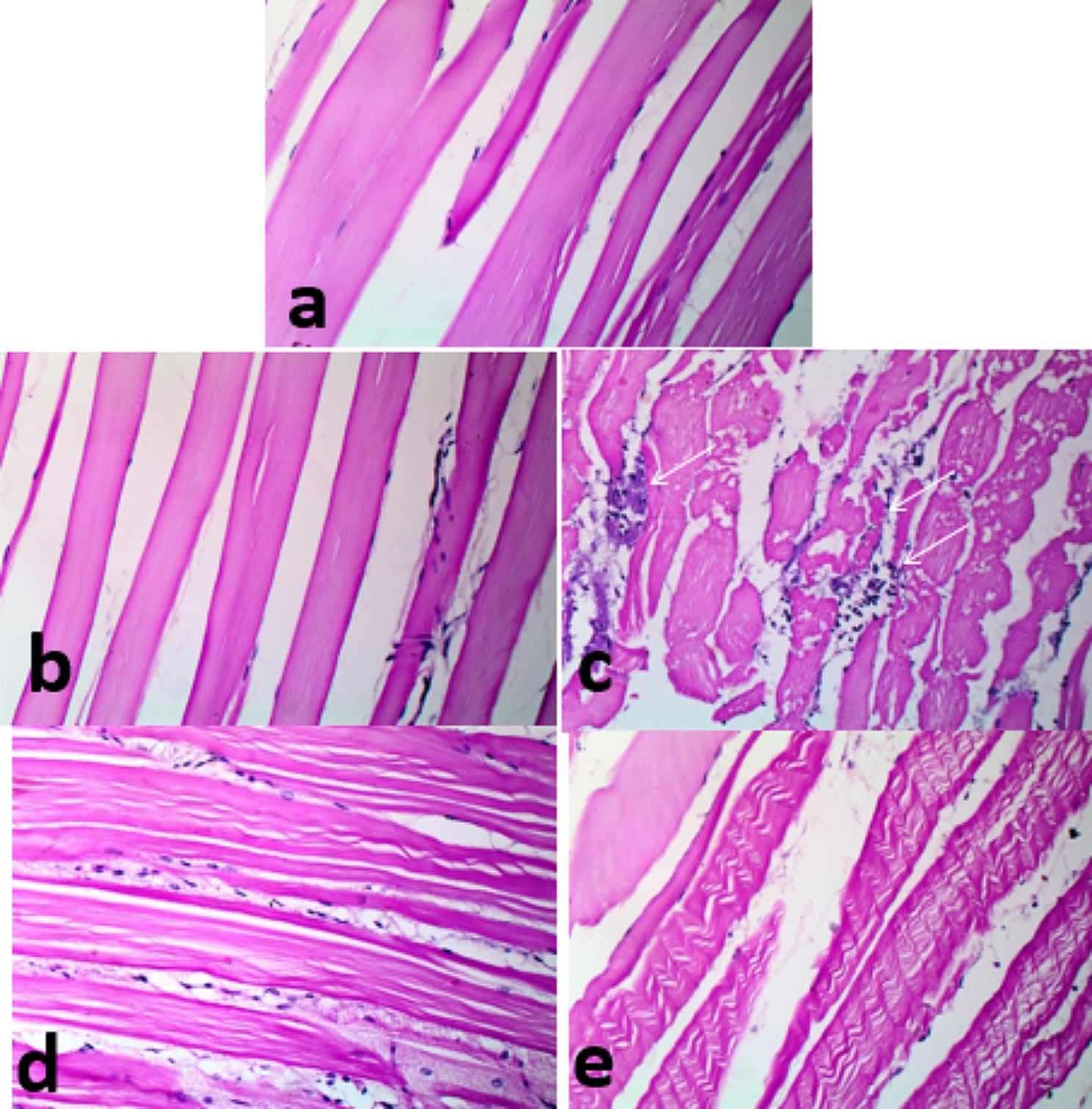
Muscle bundles; normal histological criteria of muscle bundles in (**a** & **b**). showing foci of bacteria aggregation in form of blue foci, vacoulation of muscle bundles (white arrows, **c**), Evidence of muscle regeneration in form of; increase cellularity, splitted bundles (**d**) and corrugation (**e**). *Note* (**a**); control fish, (**b**); fish received *S. platensis*, (**c**); fish control and infect with *E. tarda*, (**d**); fish received 5 g/kg fish feed *S. platensis* and infect with *E. tarda* (**e**); fish received 10 g/kg fish feed *S. platensis* and infect with *E. tarda*. H&E X 400

In Fig. 7, histological features of the gills of control fish (a), fish received 5 g (b) and 10 g (c) /kg fish feed *S. platensis* were normal. In contrast, fish of the control and infected with *E. tarda* (d) showed truncated lamellae and tips sloughing. Fish (e) received *S. platensis* and infected with *E. tarda* had lamellar aneurysm. In Fig. 8, fish in the control and fish feed *S. platensis* groups (a, b, and c) showed normal hepatic-parenchyma and development of pancreatic acini with the occurrence of some melano-macrophage cells. The hepatic tissue of control fish infected with *E. tarda* (d) showed liquifactive necrosis in the form of a wide area of lysed hepatic-parenchyma, fish received *S. platensis* and infected with *E. tarda* (e) hepatic had a small lysed area in the hepatic-parenchyma. In Fig. 9, control fish infected with *E. tarda* (d) showed marked depletion of lymphocytic elements without melano-macrophage centers, whereas some alterations were recorded in control (a), which were moderately sized melano-macrophage centers. In contrast, fish fed *S. platensis* (b) and (c) showed wide sized foci of melano-macrophage, dispersed and melano-macrophage cells. A moderate sized melano-macrophage centers in fish fed *S. platensis* and infected with *E. tarda* (e). In Fig. 10, Muscle bundles; normal histological criteria of muscle bundles in control fish (a) and fish received *S. platensis* (b). Muscle bundles of control fish infected with *E. tarda* showed foci of bacteria aggregation, vacuolation of (white arrows, c), Evidence of muscle regeneration in the form of increased cellularity, split bundles of fish received 5 g/kg fish feed *S. platensis* and infected with *E. tarda* (d) and corrugation in fish received 10 g/kg fish feed *S. platensis* and infected with *E. tarda* (e).

## Discussion

In this work, the concentration of ammonia compounds of the affected fish farm was high enough to be stressful and immune depressed for Nile tilapia, including TAN (0.98–1.29 mg/L), NH_3_ (0.11–0.15 mg/L), NO_2_ (0.04–0.06 mg/L), and NO_3_ (0.88–0.93 mg/L) despite partial replace of the pond water. In accordance, Nile tilapia that were exposed to 0.53 and 0.265 mg/L of NH_3_ and infected with *Clostridium perfringens* type A suffered from high MR 80% and 70%, respectively [49], these results could be due to impairment of the immune responses that resulted in an upsurge of the vulnerability of fish to bacterial infection [50]. Likely, the safe maximum concentration of NH_3_ is 0.1 mg/L **for** short-term in freshwater fish [51] **and** 0.012 mg/L seawater for long-term in Atlantic salmon posts molts (*Salmo salar* L.) [52].

Nile tilapia naturally and experimentally infected with *E. tarda* showed similar clinical signs and post-mortem lesions that were signs of septicemic bacterial diseases, including external hemorrhages, tail rot, ascites, splenomegaly, and inflamed intestines. Similarly, both freshwater fish *O. niloticus and Clarias gariepinus* naturally infected with *E. tarda* had skin ulcers and hemorrhages, ascites, muscular hemorrhage, congested liver with splenomegaly; these signs were similar to those observed with the experimentally infected fish [53], in addition, **Rao et al.** [54] and **Leung et al.** [55] claimed that *E. tarda* produced bacterial toxins (various virulence factors) such as hemolysin, catalase and extracellular products (ECPs) that could attack the host cells causing tissue inflammation and damage resulting in immunodepression status in fish.

In the last decades, bacterial identification has relied on genetic-based investigations to avoid the shortcomings of conventional methods [56, 57]. In this work, naturally diseased Nile tilapia were positive for *E. tarda*, PCR, and sequence of *gyrB1.* Accordingly, **Lan et al.** [56] found that the *gyrB1* gene could be used as a marker to screen the presence, identification, and classification of *Edwardseilla* species, while the *edw1* gene was used to confirm the virulence and pathogenicity of *E. tarda* [58]. Similarly, in Egypt, isolated *E. tarda* harbored *edw*I and *qse*C genes [53], and *pvs*A gene and *edw1* gene [59]. These genes *(edw*I, and *qse*C genes) are responsible for the virulence of *E. tarda* by regulating flagellar motility, biofilm formation, and quorum sensing [60].

In the indoor bacterial challenge test, we determined the LD_50_ of *E. tarda* to be 1.7 × 10^5^ CFU/mL for Nile tilapia (50 g) under specific water conditions. It is important to note that LD_50_ can vary significantly among different fish species. For instance, different LD_50_ values were detected for freshwater fish [61], African catfish and Nile Tilapia [62]. These variations can be attributed to the *E. tarda* strain, the fish’s weight and size, and accommodation conditions. This information is crucial for understanding the impact of *E. tarda* on different fish species.

We found that *E. tarda* was sensitive to Trimethoprim & Sulfamethoxazole, showed intermediate sensitivity to florfenicol, and was resistant to several other antibiotics tetracycline, ciprofloxacin, erythromycin, gentamycin, amoxicillin, ampicillin kanamycin, cefotaxime, and streptomycin. However, **Kumar et al.** [63] reported that *E. tarda* strains were multiply resistant to antibiotics, with a high percentage of isolates retrieved from aquatic animals in West Bengal and Bihar, India. These findings underscore the urgent need for further research into alternative treatment strategies, highlighting the importance of our collective efforts in this field.

In this experiment, the growth performance was enhanced in Nile tilapia (45.05 ± 0.4 g), fed *S. platensis* at doses of 5 and 10 g/kg fish feed. Similar findings with different fish species were recorded by **Adel et al.** [64], who stated that growth performance and feed utilization of great sturgeon (*Huso huso*) were improved with *S. platensis* supplementation at a dose of 100 g/kg fish feed. Also, **Mabrouk et al.** [65] had similar findings with Nile tilapia fed on dietary *S. platensis* at 10 /kg fish feed. Some authors fed fish on *S. platensis* meal and had similar outcomes. Also, **Rahman et al.** [66] found that Stinging catfish *(Heteropneustes fossilis)* fed a diet containing *S. platensis* meal (50 g/kg fish feed) obtained significantly improved WG and DWG than the control ones. Furthermore, Nile tilapia fed dietary *S. platensis* could utilize feed ingredients more efficiently, so it could be considered an essential element of Nile tilapia natural diet [67].

The importance of natural barriers, including inflammatory cytokine, in the fight against bacterial infections is well known. The pro-inflammatory cytokines *(IL-1β* and *TNF-α)* are the supporting columns of humoral cell-mediated immune responses, and they activate macrophages, production of (nitric oxide reactive oxygen species, and reactive nitrogen intermediates), and autophagy [68]. In this work, gene expression of *IL-1β* and *TNF-α* were upsurged in response to *E. tarda* infection by 3 h, with more intensity in fish that received dietary *S. platensis*. Furthermore, the expression of *IL-1β* and *TNF-α* was subsided with lower intensity after 48 h post-infection. This finding was consistent with **Mayer-Barber and Yan** [69], who claimed that *IL-1β* is a rapid cytokine that responds to acute bacterial infections; it also stimulates the secretion of chemokines that are required for the optimal control neutrophil-dependent. Likely, the immunity of Nile tilapia was significantly enhanced by feeding *S. platensis*-diets at 1% more than 2% with a rapid down-regulation of anti-inflammatory cytokine *(IL-10)* [70]. Also, **Awad et al.** [71] found that the serum of Nile tilapia received dietary *S. platensis* had a low content of *TNF-α* and *IL-10*, whereas after *A. hydrophila* infection, a significant rise in *TNF-α* and a significant downregulation in *IL-10* in control. This finding was inconsistent with our study as the infected fish (control) was immunosuppressed, and the inflammatory status persisted. Controversially, **Song et al.** [72] claimed that grass carp *(Ctenopharyngodon idella)* infected with *A. hydrophila*, intestinal pro-inflammatory cytokines *(IL-1, IL-8*, and *TNF-α)* were upregulated, and dietary *S. platensis* significantly reduced *TNF-α* and elevates *IL-10.* This difference was due to sampling time, as our samples were collected directly after 3 and 48 h post-infection.

In this experiment, MR% of Nile tilapia challenged with *E. tarda* was decreased, especially those who received 10 g *S. platensis* /kg fish feed compared with those of the control and ones who received 5 g *S. platensis* /kg fish feed, 46.67%, 60%, 60%, respectively. A 10 g *S. platensis* /kg fish feed dose could provide a 22.2% RPL. Consistent with these findings, dietary *S. platensis* had a potential immune role in different fish species, enhancing survivability and boosting the resistance against fish pathogens [64]. Similarly, African catfish and Nile tilapia injected with LD_50_ (0.2 ml of 10^4^ CFU/ml) at 25 °C resulted in MR of 70% and 60% [62]. Consistently, dietary *S. platensis* increased the resistance against *A. hydrophila* infection in African Catfish [73], *Cyprinus Carpio* [26], and Gibel carp (*Carassius gibelio*) [74]. These findings could be due to the high immune response in Nile tilapia received *S. platensis* compared to the control. Similarly, common carp *(Cyprinus carpio)* fed dietary *S. platensis* showed high cytokines generation with rapid onset [26]. Moreover, a small dose of *S. platensis* (10 mg/fish /day) could improve the general health of Nile tilapia. Also, it could stimulate the immune system, making fish more resistant to infectious diseases [75]. Also, **Tawfeek et al.** [76] claimed that dietary *Chlorella vulgaris* algae could mitigate chlorpyrifos toxicity in Nile tilapia, protecting against *Streptococcus agalactiae* infection.

Nile tilapia experimentally infected *E. tarda* in this work, drastically altering their behavior: high OM, low food apprehension, and abnormal swimming. Also, it was noticed that dietary *S. platensis* could ameliorate these withdrawals with a fast restoration of normal status. Inconsistency acute and chronic stress in aquatic animals is usually accompanied by changes in feeding behavior, respiratory activity, escaping behavior, swimming style, and abnormal behavior [77]. Similarly, Nile tilapia experimentally infected *E. tarda* with lethargy and showed abnormal swimming behavior and loss of escape reflexes [61]. These changes in fish behavior could be due to insufficient oxygen supply to tissues, increasing compensatory respiration in response to stress and anemia [78, 79].

Histopathological findings revealed gills truncated lamellae and tips sloughing lamellar aneurysm in control fish infected with *E. tarda*. Hepatic tissue of control and *S. platensis* groups showed normal. In contrast, hepatic parenchyma necrosis in the form of a wide area of lysed hepatic- parenchyma, fish received *S. platensis* and infected with *E. tarda*. The spleen of fish infected with *E. tarda* showed marked depletion of lymphocytic elements with the absence of melano-macrophage centers. Muscles showed foci of bacteria aggregation and vacuolation. Similarly, *E. ictaluri* infected farmed hybrid red tilapia *(Oreochromis sp.)* in Southeast Asia, muscles had various clumps of rod-shaped bacteria stained with hematoxylin were also visualized; the presence of cell pyknosis and karyorrhexis was commonly found in the infected spleen with increasing numbers of melanoma-macrophage centers (MMCs). Also, they added that severe blood congestion, hemorrhage, and necrotic hepatocytes were observed in the hepatic tissue [80]. In accordance, **Pirarat et al.** [81] stated that lymphoid depletion and lymphoid necrosis were noticeable in control Nile tilapia that was infected with *E. tarda*, while focal secondary lamellar fusions and severe anemia were observed only in the gills; also they added that *L. rhamnosus* (probiiotic) could mitigate impacts of experimental *E. tarda* infection. These pathological lesions resulted from toxins produced by *E. tarda* while antioxidant contents of *S. platensis* could ameliorate such damages. Accordingly, *Pseudomonas fluorescens* caused oxidative stress in Nile tilapia, causing severe tissue damage, and fish fed on *S. platensis* (1%) showed hyperplasia of melanoma-macrophage centers and hemopoietic elements *S. platensis* (2%) also showed hyperplasia of melanoma-macrophage center and more activation of hemopoietic tissue [70].

## Conclusion

Seventeen Nile tilapia out of 50 fish were infected with *E. tarda*, which harbored clinical and post-mortem signs resembling experimental fish. The level of NH_3_ was 0.11–0.15 mg/Lin fish farm, which was stressful for Nile tilapia. The bacterial strain was identified using API20 E (4,144,000) and molecular methods *E. tarda* strain and deposited in GenBank (PP449014). *E. tarda* (JALO4) was positive for *gyrB1* and *edw1* AHL-synthase, while LD_50_ was determined to be 1.7 × 10^5^ CFU/mL. *E. tarda* was sensitive to trimethoprim & sulfamethoxazole, with an MDR of 0.8, indicating a multiple antibiotic-resistant bacteria. Higher operculum movement, low food apprehension, and abnormal swimming were observed in experimentally infected fish. The expression *IL-1β* and *TNF-α* were intensively upregulated in *E. tarda-*challenged fish in *S. platensis* groups. Dietary *S. platensis* at a dose of 10 g/kg fish feed could provide a higher RPL of 22.2% Nile tilapia challenged against *E. tarda*.

### Electronic Supplementary Material

Below is the link to the electronic supplementary material.

Supplementary Material 1

## Data Availability

Data is available on request from the corresponding author.
