# Supplementary figures and images for: Overview on the role of dietary Spirulina platensis on immune responses against Edwardsiellosis among Oreochromis niloticus fish farms
Source: BMC Vet Res. 2024 Jul 4;20:290. doi: 10.1186/s12917-024-04131-7 (PMC11223423; doi:10.1186/s12917-024-04131-7)

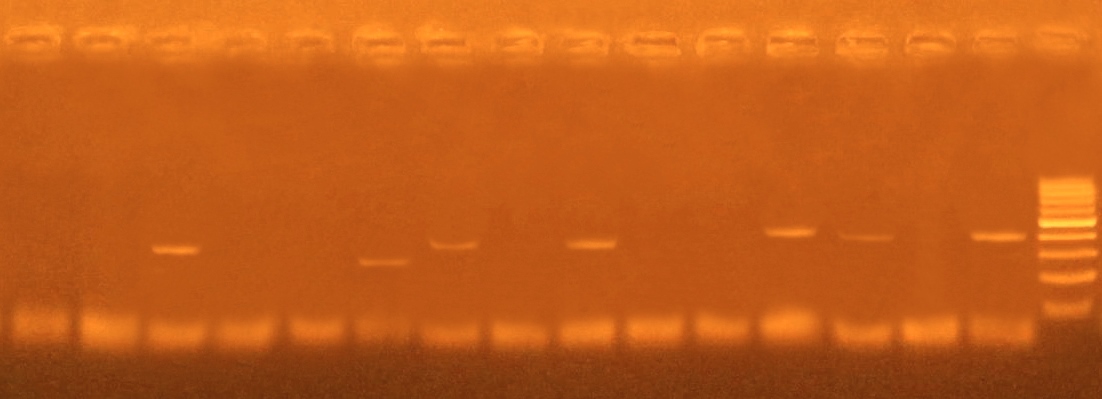

Supplement: Supplementary file 1 — Supplementary Material 1 [file 12917_2024_4131_MOESM1_ESM.jpg]
